# Perioperative Care for Bariatric Surgery

**DOI:** 10.3390/diagnostics14182095

**Published:** 2024-09-23

**Authors:** Reno Rudiman, Ricarhdo Valentino Hanafi

**Affiliations:** 1 Division on Digestive Surgery, Department of General Surgery, School of Medicine, Universitas Padjadjaran, Hasan Sadikin General Hospital, Bandung 40161, Indonesia; 2 Department of General Surgery, School of Medicine, Universitas Padjadjaran, Hasan Sadikin General Hospital, Bandung 40161, Indonesia; ricarhdo22001@mail.unpad.ac.id

**Keywords:** bariatric surgery, digestive surgery, obesity, perioperative care, postoperative care

## Abstract

This review will start with a brief pathophysiology of obesity and the requirement for bariatric surgery, and it continues with a preoperative assessment, which includes a surgical mortality risk assessment, respiratory and cardiovascular assessments, and a psychological assessment. In-hospital postoperative care will be discussed, including which patients need a surgical intensive care unit and the monitoring tools required. The need for postoperative medications, postoperative complications, strategies for management, and a follow-up plan are also reviewed. This manuscript is written in a narrative review form with a chance of bias as a possible limitation.

## 1. Introduction

The World Health Organization (WHO) defines obesity as an abnormal or excessive build-up of fat; it has been referred to as a “global pandemic”. Body mass index (BMI) measurements determine if a person is overweight or obese. An individual with a BMI exceeding 30 kg/m^2^ is categorized as obese, whereas a BMI ranging from 25 to 29.9 kg/m^2^ is classified as overweight. In certain populations, the criteria for obesity and being overweight are not the same [1].

Based on a recent analysis of data from 195 nations, the occurrence of obesity has doubled in over 70 countries since 1980. In 2015, the global population of obese individuals exceeded 600 million, and a high body mass index (BMI) was associated with 4 million mortalities worldwide [2]. The elevated morbidity and mortality rates connected to obesity are attributed to its involvement in several chronic medical ailments, such as cardiovascular and metabolic disorders, hypercoagulable states, lower back pain, osteoarthritis, and cancer. According to a recent article, the National Health Census in 2007 found that 23.1% of the Indonesian adult population had obesity and 28% had central obesity. The prevalence of both obesity criteria is higher in females than males [3].

The primary cause of obesity is an imbalance between energy expenditure and calorie consumption. Excessive energy consumption results in the accumulation of fat and glycogen in the layer of fat beneath the skin (subcutaneous adipose tissue) and in organs [4].

There are two primary patterns of fat distribution in different regions of the body: central and peripheral. Central obesity, also known as abdominal or android obesity, is defined by the accumulation of fat in the thorax, abdomen, and visceral organs. Peripheral (gynoid) obesity is defined by the accumulation of adipose tissue in the hips, thighs, and limbs, as well as in the subcutaneous layer. Central obesity, as measured by the waist circumference (WC) or the waist-to-hip ratio (WHR), has been proposed as a more effective predictor of cardiovascular disease (CVD) risk compared to utilizing solely the BMI as a measure of obesity. Presently, the WHO has established that central obesity is characterized by a WC over 102 cm in men and 88.0 cm in women. Additionally, a WHR greater than 40.8 in women and 40.9 in males is considered indicative of central obesity [5,6].

In order to prevent intraoperative and postoperative difficulties associated with these changes in obese patients’ body systems, the purpose of this manuscript is to elucidate how obesity affects the key body systems as well as the holistic perioperative assessment and postoperative management of bariatric surgery. There is also a discussion of potential postoperative complications, management techniques, and a long-term follow-up plan.

## 2. Obesity and Respiratory System

Obesity is strongly linked to respiratory symptoms and conditions such as difficulty breathing during physical exertion (exertional dyspnea), a sleep disorder characterized by interrupted breathing during sleep (obstructive sleep apnea syndrome or OSAS), a condition where obesity leads to inadequate breathing (obesity hypoventilation syndrome or OHS), a chronic lung illness that causes breathing difficulties (chronic obstructive pulmonary disease or COPD), a chronic inflammatory disease of the airways (asthma), a blockage of the lung arteries by a blood clot (pulmonary embolism), and a lung infection caused by inhaling food or liquid (aspiration pneumonia). Obesity increases susceptibility to respiratory infections, and obese patients with respiratory disease have higher rates of hospitalization compared to those who are at a healthy weight [7].

Obesity impacts the breathing pattern by decreasing the compliance of the respiratory system as a whole, including the lungs and chest wall. Because fat builds up inside the thoracic and abdominal cavities, it restricts the diaphragm’s downward movement and the chest wall’s outward movement, causing a modest increase in intra-abdominal and pleural pressures in obese people [8]. Cross-sectional and longitudinal studies have shown that an increase in BMI reduces the forced expiratory volume in 1 s (FEV1), forced vital capacity (FVC), functional residual capacity (FRC), and expiratory reserve volume (ERV). Individuals with morbid obesity (defined as having a body mass index [BMI] of more than 40 kg/m^2^) experience a slight reduction in both residual volume (RV) and total lung capacity (TLC) [9].

The hallmark of OSA is recurrent obstructive apneas caused by a collapsing upper airway (UA) during sleep. This leads to recurring episodes of low oxygen levels during the night, interrupted sleep, and excessive sleepiness during the day [10]. An individual with a BMI greater than 40 kg/m^2^ has a probability of developing OSA ranging from 55% to 90% [11]. Fat deposits around the soft tissues of the tongue and neck increase extra-luminal pressures in the pharynx, which raises the risk of airway collapse. Obesity leads to decreased lung capacities, specifically FRC and TLC, which in turn increases the pressure that causes the pharynx to close passively. Additionally, there is a higher chance of post-surgery desaturation, cardiac events, respiratory failure, and ICU admissions with this syndrome [12,13,14].

To diagnose OHS, a BMI of more than 35 kg/m^2^ (indicating clinical obesity) and daytime hypercapnia (Paco2 > 6 kPa) are necessary [15]. Adipose-derived leptin is a hormone that reduces appetite and helps stop overindulging in food, which helps stop people from gaining more weight. It has been suggested that leptin may help obese individuals maintain sufficient minute ventilation [16]. A considerable percentage of individuals who are obese exhibit increased levels of leptin, indicating the presence of leptin resistance [17]. Oxygen desaturation events that occur during the night are typically more severe and frequent in individuals with obesity hypoventilation syndrome (OHS), leading to more intense levels of low oxygen levels and high carbon dioxide levels throughout the night. This can lead to increased sympathetic activation, heightened oxidative stress, and higher cardiovascular risk factors. In patients with OHS, persistent hypoxemia, and hypercapnia during the day are linked to an increased risk of pulmonary hypertension, cor pulmonale, right-sided heart failure, and post-surgery respiratory arrest [15].

## 3. Obesity and Cardiovascular System

Obesity is also a direct cause of cardiovascular disease and death from cardiovascular disease, regardless of other risk factors for cardiovascular problems [18]. Imaging studies have demonstrated a strong association between visceral obesity and increased deposition of fat in the liver [19]. Additional ectopic fat deposits that are of significance include the adipose tissues around the heart, known as pericardial and epicardial adipose tissues [20]. There is a correlation between the thickness of epicardial adipose tissue and waist circumference, blood pressure, indicators of insulin resistance, and dyslipidemia. A study by Shetty et al. showed a strong, positive correlation between the epicardial fat thickness obtained from echocardiograms, with waist circumference, and an increase in blood pressure that is associated with arterial stiffness [21].

Obesity expedites the early development of atherosclerotic alterations by insulin resistance and inflammation. Inflammation enhances the probability of the oxidation of low-density lipoprotein, hence facilitating the development of atherosclerosis. Insulin resistance is connected to dyslipidemia, which includes elevated triglycerides, reduced levels of high-density lipoprotein cholesterol, and the presence of small, dense low-density lipoprotein particles. These factors are also related to metabolic syndrome, and together they contribute to the development of atherosclerosis [22]. Obesity is a significant risk factor for hypertension, CVD, and left ventricular hypertrophy. These conditions are all strong risk factors for the development of HF. Based on a study involving 5881 participants from the Framingham Heart Study, it was observed that the incidence of HF increased by 5% in men and 7% in women with every one-unit rise in BMI [23]. The excessive buildup of fat increases the circulating blood volume and the release of inflammatory substances that promote the development of atherosclerosis. These factors contribute to an increase in stroke volume, cardiac wall stress, and damage to the heart muscle, resulting in the development of concentric left ventricular hypertrophy (LVH), the remodeling of the left ventricle, and ultimately, diastolic and systolic HF [24].

A BMI between 30 and 34.9 kg/m^2^ is linked to a 54% higher chance of transitioning from paroxysmal to persistent atrial fibrillation (AF) [25]. Obesity leads to a gradual restructuring of the atria, characterized by an increased accumulation of fibrous tissue, a heightened expression of endothelin receptors, and irregularities in atrial conduction. As a result, this results in a relative risk of 1.5 for atrial fibrillation and a significantly elevated risk of sudden cardiac death [26].

## 4. Obesity and Thrombosis

Obesity raises the probability of developing thrombotic disorders such as myocardial infarction, stroke, and venous thromboembolism (VTE), leading to higher rates of illness and death. Obese women experience a postoperative occurrence of VTE that is ten-fold more than that of women who have a normal body weight. Patients with a history of VTE are at a specific risk when undergoing gastric bypass surgery [27]. The two main metabolic problems associated with obesity that are most likely to cause obesity-induced thrombosis are decreased fibrinolysis and chronic inflammation [28].

Adipocytes generate inflammatory cytokines that cause chronic, low-grade inflammation. This inflammation then attracts macrophages to the adipose tissue. Proinflammatory cytokines stimulate the vascular endothelium, platelets, and other circulating vascular cells. This stimulation causes an increase in procoagulant factors and adhesion molecules, a decrease in anticoagulant regulatory proteins, an increase in thrombin generation, and an enhancement of platelet activation. Inflammatory cytokines also induce the production of adhesion molecules, such as P-selectin, which facilitate contacts between endothelial cells and leukocytes, as well as between platelets and leukocytes. This process further enhances the formation of blood clots [29]. Fibrinolysis is a vital physiological mechanism that leads to the prompt breakdown of the fibrin clot by plasmin. Plasminogen activator inhibitor-1 (PAI-1), a serine protease inhibitor, is released by the vascular endothelium, the liver, and adipose tissue to highly regulate the pace of fibrinolysis. Individuals with a higher BMI and WHR exhibit heightened levels of PAI-1 [30].

## 5. Obesity and Type II Diabetes Mellitus

Obesity increases the risk of developing type 2 diabetes mellitus (T2DM). The Second Diabetes Surgery Summit (2016) created a therapeutic protocol for metabolic and bariatric surgery in individuals who have both obesity and diabetes [31]. Bariatric surgery led to a five-fold reduction in T2DM incidence over 7 years [32]. The chance of developing diabetes throughout a lifetime in men over the age of 18 increases significantly from 7% to 70% when their BMI climbs from less than 18.5 kg/m to more than 35 kg/m. Likewise, the likelihood of developing diabetes over one’s lifetime in females rises from 12% to 74% when considering the same BMI values [33].

Adipose tissue affects metabolism by the release of hormones, glycerol, and other substances including leptin, cytokines, adiponectin, and proinflammatory chemicals. Additionally, it releases non-esterified fatty acids (NEFAs). In obesity, there will be an elevated secretion of these chemicals. Elevating the concentration of NEFA in the bloodstream leads to the development of insulin resistance [34]. Furthermore, there is also a decrease in the regulation of β-cell function. Prolonged exposure to NEFAs is associated with notable dysfunction in pathways responsible for glucose-stimulated insulin secretion and decreased insulin production, which may result in increased morbidity and poor glycemic control during perioperative periods [35].

## 6. General Consideration for Pre-Bariatric Surgery Preparation

Patients who are at an increased likelihood of encountering complications after surgery include those who have central obesity and metabolic syndrome, as opposed to those who solely have excessive obesity [36]. The Obesity Surgery Mortality Risk Stratification score (OS-MRS) has been verified to identify the risk factors linked to mortality (Table 1 and Table 2). While this has only been proven to be true for individuals receiving bariatric surgery, it could also be relevant for obese patients who are having non-bariatric procedures. Patients with an OS-MRS score of 4–5 are more prone to necessitate more intensive postoperative surveillance [37].

Before bariatric surgery, a “Liver Shrinking Diet” (LSD) is usually started to shrink the liver, facilitate easier stomach access, and reduce the risk of bleeding. According to the research, a two-week to four-week reduced calorie diet before surgery led to a notable reduction in liver size and fat accumulation within the liver. LSDs involve approximately 800–1000 kcal intake per day with less than 100 g of carbohydrate, low fat, and high protein. An LSD may either be a milk yogurt diet or a food-based diet [38]. Prior to surgery, engaging in a conversation with the patient can help encourage them to quit smoking, explain the significance of preventing blood clots and starting physical activity early, and establish a plan for managing medication prior to admission [39].

## 7. Respiratory Assessment for Pre-Bariatric Surgery Preparation

Obesity is linked to a 30% higher likelihood of having trouble or failure in the process of intubation. However, the factors that predict problematic laryngoscopy are the same for both obese and non-obese individuals. A substantial neck circumference is a valuable supplementary signal, and if it exceeds 60 cm, it is linked to a 35% likelihood of encountering challenges during laryngoscopy. Bag-mask ventilation is also more challenging in those who are obese [40]. Spirometry should be used to clinically evaluate the respiratory system and exercise tolerance in order to identify any functional limitations and provide guidance for future examination. The presence of the following signs may suggest the existence of substantial underlying respiratory disease and should lead to consideration of preoperative arterial blood gas analysis [41]:

- Arterial saturation < 95% on air;
- Forced vital capacity < 3 L or forced expiratory volume in 1 s < 1.5 L;
- Respiratory wheeze at rest;
- Serum bicarbonate concentration > 27 mmol/L.

The Snoring, Tiredness, Observed apnea, high blood Pressure (STOP) and Body mass index, Age, Neck Circumference, and Gender (BANG) screening tool has been extensively validated in surgical patients (Table 3) [42]. For individuals who are obese, a STOP-BANG score greater than 3 has a sensitivity above 90% in identifying OSA, with a positive predictive value of 85%. A score greater than 5 has a sensitivity of 53% and a specificity of 70% in predicting moderate to severe OSA [43]. The American Society of Anesthesiologists Task Force on Perioperative Management of Patients with OSA advises that patients with confirmed or suspected OSA should undergo a preoperative assessment, which includes sleep studies, patient interviews, and physical examinations. An in-depth interview should cover topics such as snoring, episodes of apnea, frequent awakenings during sleep, morning headaches, and daily sleepiness. The physical examination should include an evaluation of the airway, nasopharyngeal structure, neck circumference, and tongue size. Patients with confirmed OSA may receive preoperative preparation before surgery, which may include the utilization of continuous positive airway pressure (CPAP) or bilevel positive airway pressure (BiPAP) devices. Additionally, a preoperative oral appliance may be used to minimize postoperative problems such as increased carbon dioxide levels (hypercarbia), low oxygen levels (hypoxemia), and constriction of the pulmonary artery [44].

## 8. Cardiovascular Assessment for Pre-Bariatric Surgery Preparation

It is necessary to acquire a comprehensive preoperative cardiac assessment, which includes a thorough examination of the patient’s medical history, physical condition, and functional capacity. An individual’s functional status can be deduced by assessing their capacity to carry out tasks that are part of their daily routine. Moreover, a higher level of cardiorespiratory fitness is linked to a reduced likelihood of experiencing negative cardiac events. An evaluation of cardiac risk should be conducted according to the standards provided by the American Heart Association (AHA) [45]. Essentially, the decision to perform more cardiac tests depends on assessing the patient’s risk factors, evaluating the heart risk related to the scheduled surgery, and reviewing the patient’s functional status. The American College of Surgeons NSQIP Risk Calculator and the Revised Cardiac Risk Index are reliable tools that have been proven to accurately estimate the risk associated with surgical procedures [46].

ACE inhibitors and ARBs may have a positive effect due to their capacity to enhance insulin sensitivity [47]. Pulmonary hypertension patients should undergo a preoperative assessment, which comprises an electrocardiogram (ECG) and an echocardiogram. These tests are utilized to assess the morphology and functionality of the ventricles and valves. It is crucial to engage pulmonary and/or cardiac experts in this evaluation [48].

## 9. Psychiatric Assessment for Pre-Bariatric Surgery Preparation

The purpose of the presurgical psychological evaluation goes beyond diagnosing any mental health conditions to include assessing patients’ mental health stability before surgery, their level of motivation for the procedure, and their degree of adherence to presurgical lifestyle changes (diet, exercise, etc.). A clinical interview and psychological testing are the two components of a psychological assessment conducted by a psychologist or mental health professional (MHP) [48].

To assess a person’s personality difficulties, such as their degree of impulsivity, binge-eating disorders, comorbid depression, anxiety disorders, etc., a clinical interview should be undertaken. This includes reviewing the patient’s weight and dietary history, including at which point weight became an issue, the kinds of diets attempted, the results of those attempts, the reasons for weight gain, and any family history of obesity. Most patients have a long history of trying diets with minimal long-term results. Approximately 10–25% of the patients fit the criteria for binge-eating disorder, which is characterized by consuming a lot of food in a short amount of time—less than two hours—and experiencing a subjective loss of control. In order to successfully maintain their weight over the long term, patients are also asked about their attitudes, knowledge, and future exercise goals in their daily routine [49].

Psychological testing should be performed (Beck Depression Inventory, the Minnesota Multiphasic Personality Inventory, Binge Eating Scale, and Millon Behavioral Medicine Diagnostic). Following the collection and scoring of all this data, a report is summarized for the surgeon in order to decide whether or not the patient is a good candidate for bariatric surgery. Surgery should be postponed or specific recommendations for intervention before surgery may be suggested if the psychologist determines that the patient lacks the capacity (to understand the risks, benefits, and results of the surgical procedure; a reluctance to adhere to the postoperative recommendations) or has certain psychiatric illnesses [50].

## 10. Preoperative Preparation Check List

The preoperative preparation checklist includes the following [51]:

- Preoperative general medical check-up;
- Liquid diet starts from 5 days preoperative;
- NPO 6 h preoperative;
- Prophylaxis antibiotics 1 h preoperative;
- Leg stocking preoperative.

## 11. Intraoperative Possible Challenges and Management

Because obesity promotes the uncontrolled lipolysis of visceral fat that is rich in unsaturated triglycerides, which results in necrosis, obesity exacerbates acute pancreatitis. Significant adhesions in the smaller sac caused by pancreatitis may make it technically challenging to construct the gastric pouch during gastric bypass surgery. Additionally, there is a chance that inflammation will cause pancreatic damage or develop a pancreatic fistula. In this situation, LSG might be a better option than a gastric bypass [52].

Studies have shown that 1–5% of individuals may experience unexpected macroscopic symptoms of liver cirrhosis during bariatric surgery. This disorder is linked to a higher risk of bleeding during any kind of surgery due to liver function abnormalities and portal hypertension. In this condition, LSG is the safest option for bariatric surgery for patients with compensated cirrhosis who do not have a high level of portal hypertension. During the informed consent process, surgeons should discuss with all bariatric patients the likelihood of unanticipated intraoperative symptoms of liver cirrhosis and work out a different plan of action if necessary [52].

The diaphragmatic hiatus, the gastro-oesophageal junction, and the angle of His may be unsafe to observe or access if there is significant hepatomegaly, splenomegaly, or both. Thus, there is a chance of esophageal, hepatic, and splenic damage when doing a laparoscopic gastric bypass in this situation. Because gastric bypass has a higher risk of difficulties due to the small amount of space available for a safe gastrojejunal (GJ) anastomosis, an LSG procedure is recommended [52].

Furthermore, patients with a BMI over 50 kg/m^2^ have high rates of OSA, cardiac insufficiency, hypertension, and diabetes and are strongly linked to respiratory and heart failure, as well as extubation failure under anesthesia. Highly experienced anesthetists are required. Tracheal intubation is made easier by having the patient lie down with a pillow under their head, aligning their upper sternum with the external auditory canal, and utilizing a video laryngoscope. An EKG, pulse oximeter, non-invasive arterial blood pressure monitor, and muscle relaxation monitor should be used to monitor patients while they are under anesthesia. Preoxygenation should be performed for at least 3 min [53].

## 12. In-Hospital Postoperative Care

Continuous monitoring must be sustained in the post-anesthesia care unit (PACU). The patient should be positioned in an upright sitting posture or with a 45° head-up tilt. Administering oxygen therapy is necessary to maintain the preoperative levels of arterial oxygen saturation. This treatment should be continued until the patient is able to move around after the surgery. Patients with a history of cardiac arrhythmias or coronary artery disease, as well as those with COPD, sleep apnea, and/or asthma, are advised to undergo continuous monitoring of their heart and oxygen saturation levels over a period of 24 to 48 h. Prior to transferring obese patients to the main surgical ward, it is necessary to monitor them in the PACU for a minimum of 1 h without any external stimulation. This observation is conducted to identify signs of hypoventilation, such as a decrease in oxygen levels or instances of interrupted or shallow breathing accompanied by a decrease in oxygen levels. Surgical Intensive Care Unit (SICU) monitoring is typically unnecessary unless the patient has complex or unresponsive coexisting medical conditions or the surgery involved significant blood loss, unstable heart function, or extended use of a breathing tube. The patient may safely return to the ward only under the following conditions: [54]

- Routine discharge criteria are met;
- The respiratory rate is normal, and there are no periods of hypopnea or apnea for at least one hour;
- The arterial oxygen saturation returns to the preoperative values with or without oxygen supplementation.

NPO and IV liquids are commonly administered to patients during the initial 24 h after surgery. During this time, the amount of urine produced is observed to assess fluid balance, and tests are conducted to measure electrolyte levels and renal function as well as a complete blood count. Rhabdomyolysis is an infrequent yet severe condition that can occur in obese individuals. It is important to rapidly evaluate the concentration of serum creatinine kinase. If it is increasing, vigorous fluid resuscitation, diuretics, and urinary alkalinization may be necessary to prevent additional acute renal injury [55].

The National Institute for Health and Care Excellence issued guidelines for postsurgical VTE prophylaxis in 2010. Methods to mitigate the likelihood of VTE encompass prompt mobilization after surgery; the employment of mechanical compression devices; the utilization of thromboembolic device (TED) stockings; the administration of anticoagulant medications; and the implementation of vena cava filters. Anticoagulant administration is the primary approach for preventing VTE in obese individuals. Indications for anticoagulant administration are extended periods of immobility, surgical procedures lasting more than 90 min, individuals over the age of 60, a BMI over 30 kg·m^−2^, the presence of malignancy, dehydration, and a family history of VTE [56]. As warfarin’s anticoagulant effect can be routinely monitored by INR, this seems to be the preferred agent to use in patients after bariatric surgery. Drug disposition depends on the food volume, pH, transit time, gastrointestinal absorptive surface, and location of drug absorption, which may all affect PK and the bioavailability of an ingested drug. Thus, post-bariatric patients require dose adjustments of warfarin ranging from 7.7 mg/week, with a decrease on days 8–14 after surgery, to 30 mg/week, with a decrease on days 50–56. Overall, the literature suggests that warfarin dosing is reduced in the immediate postoperative period (within 3–4 weeks), with a trend toward increased dose requirements as patients are further out from surgery. If direct oral anticoagulants (DOACs) are used in a patient after bariatric surgery, it is suggested to check drug-specific peak and trough levels. Anticoagulants should be administered for at least 3–6 months [57]. Overby et al. classify high-risk patients as those who required a prophylaxis inferior vena cava filter (IVC) for extreme obesity (BMI over 50 kg·m^−2^) or had clinical or laboratory evidence of a thrombophilia, poor ambulation, severe sleep apnea with obesity hypoventilation syndrome, or a history of DVT or PE [58].

For postoperative patients who need intravenous opioids, it is advisable to use patient-controlled analgesia (PCA), using drugs like fentanyl or morphine, rather than a continuous infusion. It is important to also take into account a longer duration of time between doses than what is often recommended. The administration of pain relief through the enteral route should be initiated as soon as it is feasible [59].

## 13. Postoperative Care: Discharge

Enhanced recovery after surgery (ERAS) has resulted in the establishment of standards for effective perioperative treatment in bariatric and metabolic surgery. This encompasses prehabilitation, a comprehensive strategy for pain management and the prevention of postoperative nausea and vomiting (PONV) as well as early mobilization following surgery. Implementing ERAS protocols in bariatric and metabolic surgery is both secure and practical, successfully reducing the duration of hospitalization without compromising the occurrence of complications and expediting the recovery of patients. Research has demonstrated that patients who undergo Laparoscopic Adjustable Gastric Binding (LAGB) can safely be discharged on the first day after surgery. Patients who undergo Roux-en-Y Gastric Binding (RYGB) or Sleeve Gastrectomy (SG) can be discharged on the second day after surgery, or earlier if they are able to tolerate clear fluids. Patients who undergo an open procedure may be discharged as late as the fifth day after surgery [60].

## 14. Postoperative Care: Patient Education, Nutritional Guidance, and Follow-Up

Providing patient education is crucial at this point, as patients will undergo significant alterations in their bodily systems. During the initial stages following surgery, it is common to encounter symptoms such as fatigue, nausea, vomiting, insomnia, postoperative pain, weakness, dizziness, reduced appetite, flatulence, abdominal discomfort, loose stools, and fluctuating emotions. Patients may experience these symptoms to different extents. Some patients report experiencing pain at the incision site or neck and shoulder pain, which arises because of the body reabsorbing the gas utilized during surgery. It is imperative for medical assistants to administer pain management drugs during this time [61].

To significantly reduce the time it takes to recover, the most efficient approach is to engage in physical activity, such as walking short distances and regularly changing positions in bed, to enhance blood flow. The patient is advised to gradually increase their walking distance and frequency, aiming for at least four sessions per week, with each session lasting 30–45 min. This should be achieved by the end of the sixth week. Patients experiencing issues with weight-bearing joints, such as the ankles, knees, and hips, can engage in aquatic activities once their abdominal incisions have fully healed, usually around three to four weeks after the surgery. Optimal blood circulation promotes the process of healing and inhibits the development of blood clots. Avoid engaging in vigorous physical activities for a period of three to six weeks after the surgery. Refrain from lifting objects weighing more than 15 to 20 pounds throughout the initial six-week period [61].

The patient’s diet will gradually improve after they are able to accept clear liquids throughout phase 1. If the patient has tolerated clear liquids well, their diet will advance to more viscous liquids (phase 2) for the following two weeks and then to soft or pureed foods (phase 3) for a duration of 10 weeks. Phase 3 comprises “fork-tender meats” and other dietary options suitable for individuals without teeth. The commencement of a regular diet (phase 4) is often recommended approximately three months post-surgery. To reduce gastric bloating, it is advisable to abstain from consuming concentrated sugary beverages and carbonated drinks and using straws for drinking liquids. Vomiting, dysphagia, or regurgitation are abnormal outcomes of bariatric surgery and should be thoroughly examined. These symptoms might potentially arise from any type of procedure. Patients experiencing persistent vomiting should be directed to a bariatric surgery facility and are susceptible to thiamine shortage [62].

In 2020, the British Obesity and Metabolic Surgery Society (BOMSS) developed an updated nutritional guidance specifically for those who have undergone bariatric surgery. Following bariatric surgery, patients become more prone to nutritional deficiencies and require regular nutritional monitoring and the consistent use of routine nutritional supplements on an annual basis. The administration of vitamin and mineral supplements is necessary within the initial 30 days following surgery [63]:

- RYGB: vit B12 500 micrograms daily, vit C 500 mg daily;
- SG: vit B12 and iron supplement;
- RYGB and SG: vit D3 2000 IU daily.

Patients who undergo bariatric surgery require lifelong follow-up, with the specific regimen varying among different bariatric surgeons. Follow-up entails the surveillance of body weight and adherence to the postoperative regimen. Subsequent appointments, in the absence of any postoperative difficulties, are scheduled for two and four weeks after the operation. This is followed by appointments at three, six, nine, twelve, eighteen, and twenty-four months and annually thereafter. Indicators of possible problems that require prompt evaluation include body temperature above 38 degrees Celsius, intense abdominal pain, inflammation around surgical cuts, discharge from surgical cuts, continuous vomiting, chest pain, difficulty breathing, intense pain, and warmth or redness in the calf [64].

Almost a third of bariatric surgery patients present with suboptimal weight loss or important weight regain in the first five postoperative years. While the reasons underlying this are not fully understood, it is known that pathological eating styles (such as emotional or binge eating) can thwart efforts to maintain weight loss. Women displayed a higher frequency of emotional eating as compared to men, while men showed a higher frequency of a quantitative eating behavioral style [65]. The most (41%) probable reason for noncompliance following bariatric surgery was found to be a lack of exercise. Thus, a routine follow-up with a psychologist or MHP is as important as a routine medical follow-up [65].

## 15. Potential Complications of Bariatric Surgery

Possible complications that may occur include immediate intraoperative complications, late complications, and metabolic issues. According to the US Bariatric Outcomes Longitudinal Database, the 1-year complication rates for adjustable gastric band (LAG) placement, sleeve gastrectomy (LSG), Roux-en-Y gastric bypass (RYGB), and biliopancreatic diversion (BPD) were reported as 4.6%, 10.8%, 14.9%, and 25.7%, respectively [66]. The risk factors associated with an increased rate of morbidity and mortality include older age, male gender, a very high BMI, the presence of coexisting chronic diseases, the qualifications and experience of the surgeons, the qualifications and experience of the center and its available facilities, and surgeries performed using the open approach rather than the laparoscopic approach [67].

In general, the total mortality rate within 30 days after surgery is demonstrated to be less than 1%. Early complications of the procedure include surgery leak, wound infection, hemorrhage, deep venous thrombosis, and pulmonary embolism. Pulmonary embolism and surgical leak are the primary causes of death. Delayed complications related to the surgical procedure include stomal stenosis, marginal ulcers, cholelithiasis, internal and incisional hernias, short bowel syndrome, nutritional deficiencies, and dumping syndrome [68].

Metabolic problems that can arise from bariatric procedures encompass metabolic acidosis and/or alkalosis as well as electrolyte imbalances, such as low levels of calcium, potassium, magnesium, salt, and phosphorus. These imbalances have the potential to lead to arrhythmias and/or myopathies. Various nutritional abnormalities have been documented, including deficiencies in fat-soluble vitamins A, D, E, and K as well as iron and folic acid. A negative calcium balance and vitamin D deficiency can lead to secondary hyperparathyroidism, oxalosis, and kidney stones. Other deficiencies include thiamine and vitamin B12. Increased bacterial overgrowth can cause nocturnal diarrhea and abdominal distension [68].

An Indian multicenter study conducted by Goel et al. revealed that the predominant problems observed were postoperative hemorrhage (0.75%) and nutritional deficits (0.75%), followed by leaks (0.43%). A subgroup analysis revealed a notable increase in leaks and gastroesophageal reflux disease (GERD) following SG, intestinal obstruction after RYGB, marginal ulcer after one anastomosis gastric bypass (OAGB), and nutritional deficiencies after single anastomosis duodenoileal bypass with sleeve (SADI-S) [69].

Pablo et al. did a meta-analysis of 26 studies to assess early complications following bariatric surgery. These complications include anastomotic or staple line leaks, gastrointestinal bleeding, and intestinal obstruction. Anastomotic or staple line leaks are characterized by insufficient tissue healing, which results in the escape of gastrointestinal contents through the staple or suture line. Many anastomotic leaks often happen between five to seven days following surgery and are believed to be associated with ischemia. However, around 95% of anastomotic leaks that occur within two days of surgery are likely due to technical mistakes. The notion of treating leaks involves promptly identifying and addressing them. Various therapies can be provided based on the patient’s clinical condition and the severity of the leak, ranging from minimally invasive procedures to surgical reoperation. Patients who are not experiencing sepsis and have stable hemodynamics are administered intravenous antibiotics, monitoring secretions through drains, naso-enteral nutrition, or total parenteral nutrition. If the leak is contained and accessible, a percutaneous procedure can be carried out. However, if the patient is experiencing hemodynamic instability, has a complex leak, or shows signs of sepsis, surgical intervention is necessary to verify and rectify the leakage, extract gastrointestinal contents from the abdominal cavity, and insert closed suction drains [70].

## 16. Conclusions

Obesity has extensive effects on various bodily functions, particularly on the respiratory and cardiovascular systems. Given the widespread occurrence of obesity, it is necessary to adopt a cautious approach when considering many aspects of medical practice related to obesity, such as presurgical evaluation, surgical care, and postoperative procedures. The take-home message for clinicians is to provide systematic and comprehensive support for treating obese patients who have surgery in a logical manner. A holistic approach includes considering health problems related to obesity, prophylaxis treatment to prevent intra- and postoperative complications, and correct postoperative management and education to ensure favorable patient outcomes. This requires a multidisciplinary team, which includes respiratory and cardiovascular specialists, nutritionists, as well as psychiatrists. Moreover, to provide effective and comprehensive healthcare, it is crucial to thoroughly examine the consequences of obesity in the unique context of the patient, considering its complex character.

The limitation of this manuscript is a potential risk of bias, taking into account that this manuscript is written in the form of a narrative review. The author recommends further study to conduct a systematic review study regarding this topic.

## Figures and Tables

**Table 1:** The risk factor of the Obesity Surgery Mortality Risk Stratification score.

Risk Factor	Score
BMI > 50 kg/m^2^	1
Male	1
Age > 45 years	1
Hypertension	1
Risk factors for pulmonary embolism:	1
Previous venous thromboembolism	
Vena cava filter	
Hypoventilation (sleep-disordered breathing)	
Pulmonary hypertension	

**Table 2:** The risk of mortality of the Obesity Surgery Mortality Risk Stratification score.

	Risk of Mortality
Class A: 0–1 points	0.2–0.3%
Class B: 2–3 points	1.1–1.5%
Class C: 4–5 points	2.4–3.0%

**Table 3:** The Snoring, Tiredness, Observed apnea, high blood Pressure (STOP) and Body mass index, Age, Neck Circumference, and Gender (BANG) Screening Questionnaire.

	Questions
Snoring	Do you snore loudly (louder than talking or heard through a closed door?)
Tired	Do you often feel tired, fatigued, or sleepy during the daytime? Do you fall asleep in the daytime?
Observed	Has anyone observed you stop breathing or choking or gasping during your sleep?
Blood Pressure	Do you have, or are you being treated for, high blood pressure?
BMI	BMI > 35 kg/m^2^
Age	Age > 50 years
Neck	Circumference (measured around Adam’s apple) > 43 cm (17 in) for males, >41 cm (16 in) for females
Gender	Male

## Data Availability

All data are available within this study.
